# Effects of Late Spring Coldness and Recovery Light Intensity on Photosystem II and Photosystem I Function in *Mosla chinensis* Maxim. cv. Jiangxiangru Seedlings

**DOI:** 10.3390/plants15192982

**Published:** 2026-09-30

**Authors:** Yankuan Zhao, Xinqian Wang, Min Zhang, Wenhai Hu

**Affiliations:** 1 School of Life Sciences, Jinggangshan University, Ji’an 343009, China; zhaoyankuan2@jgsu.edu.cn (Y.Z.); wangxinqian5056@163.com (X.W.); 15234744435@163.com (M.Z.); 2 Key Laboratory of Jiangxi Province for Biological Invasion and Biosecurity, Jinggangshan University, Ji’an 343009, China

**Keywords:** *Mosla chinensis* Maxim. cv. Jiangxiangru, late spring coldness, photosystem II function, photosystem I function, photoprotection, chlorophyll fluorescence

## Abstract

Late spring coldness (LSC) threatens the spring cultivation of the geo-authentic (Daodi) medicinal herb *Mosla chinensis* Maxim. cv. Jiangxiangru (McJXR). This study examined the effects of LSC and subsequent recovery light intensity on photosystem II (PSII) and photosystem I (PSI) function in McJXR seedlings. The experiment was conducted during a natural LSC event in the spring of 2025. After LSC, seedlings were subjected to two recovery light regimes: the shading (R_LL_, daily maximum light intensity of approximately 500 µmol·m^−2^·s^−1^) and the full-sunlight (R_HL_, daily maximum light intensity of approximately 1100 µmol·m^−2^·s^−1^) treatments. Chlorophyll fluorescence parameters of PSII and PSI were measured synchronously at various stages using a Dual-PAM-100/F. The results showed that during the LSC period, the maximum photochemical efficiency of PSII (Fv/Fm) and the maximum P700 signal (Pm) showed no significant changes relative to the pre-stress levels (LSC-0d). By contrast, the photochemical quantum yields of PSII (Y(II)) and PSI (Y(I)) declined continuously, decreasing by 61.0% and 48.6%, respectively, by the fourth day of LSC (LSC-4d) compared with the LSC-0d levels. On the first day of LSC (LSC-1d), the photochemical quenching coefficient (qP), the maximum electron transport rate through PSII (Jmax-ETR(II)), and that through PSI (Jmax-ETR(I)) dropped by 51.9%, 70.1%, and 61.1%, respectively, and subsequently remained stable, non-photochemical quenching (NPQ) and the quantum yield of regulated energy dissipation in PSII (Y(NPQ)) increased sharply to 4.4-fold and 4.9-fold of the LSC-0d levels and remained elevated thereafter. Cyclic electron flow (CEF) showed no significant change during the LSC period. During recovery, Fv/Fm decreased under both shading and full sunlight, with a greater decline under full sunlight. However, the recovery of Y(II), qP, Jmax-ETR(II), Y(I), and Jmax-ETR(I) was greater under full sunlight than under shading, and CEF increased markedly under both treatments. By the third day of recovery, Y(I) had fully recovered to the LSC-0d level under both treatments, whereas Y(II) had not. In conclusion, the natural LSC event did not induce photoinhibition of either photosystem, but only a reversible downregulation of their photochemical activity, which was more pronounced for PSII than for PSI. The seedlings relied on photoprotective pathways, including downregulation of PSII activity and enhanced thermal dissipation, to protect the photosystems from irreversible photodamage under LSC. During recovery, the full-sunlight treatment accelerated the recovery of the photochemical activity of both photosystems relative to the shading treatment, although it also increased the degree of PSII photoinhibition; the recovery of PSI was superior to that of PSII, and the rapid decline in thermal dissipation together with the marked increase in cyclic electron flow during recovery may explain this difference. These findings contribute to understanding the responses of the two photosystems of McJXR seedlings to LSC stress.

## 1. Introduction

*Mosla chinensis* Maxim. cv. Jiangxiangru (McJXR), a cultivated variety of *M. chinensis*, is a geo-authentic (“Daodi”) medicinal herb of Jiangxi, China [1,2]. The dried aerial parts of McJXR, which are rich in thymol and carvacrol, have long been used in traditional Chinese medicine for their diaphoretic, summer-heat-clearing, and dampness-resolving properties [2,3]. McJXR thrives in warm, humid, and well-illuminated environments, and its commercial cultivation is mainly concentrated in Fenyi County, Xinyu City, Jiangxi Province [3]. Jiangxi has a typical subtropical monsoon climate, and meteorological records indicate that late spring coldness (LSC) events occur frequently, with mild and moderate events having become more frequent in recent decades, posing a significant risk to spring crops [4]. Because the seedling stage of McJXR coincides with the peak period of LSC, the seedlings face a considerable risk of chilling injury during spring cultivation.

Photosynthesis provides the foundation for crop growth and yield formation and is highly sensitive to chilling [5,6]. LSC inhibits photosynthesis mainly by disrupting the structure and function of the photosynthetic apparatus [5,7]. In wheat (*Triticum aestivum*), LSC induces marked alterations in chloroplast ultrastructure, including loosened grana lamellae, disordered thylakoid arrangement, and even loss of the chloroplast envelope with leakage of its contents [8]. LSC also severely impairs photosynthesis in rice (*Oryza sativa*) [9]. Moreover, the capacity for compensatory growth after stress differs markedly among early rice genotypes, with strong-compensating genotypes recovering rapidly through upregulation of antioxidant enzyme activities and modulation of hormone levels [4]. For medicinal plants, however, direct evidence from LSC events remains scarce. In one of the few available studies, spring chilling and freezing impaired the photochemical efficiency of photosystem II (PSII) of *Panax ginseng* [10].

Chilling suppresses carbon assimilation, generating an excess of excitation energy that threatens the integrity of the photosynthetic apparatus [5]. In the thylakoid membrane, the photosynthetic electron transport chain consists of PSII and photosystem I (PSI) arranged in series, and coordination between the two photosystems is essential for efficient electron transport [11]. PSII has long been considered the primary target of chilling-induced photoinhibition, as the D1 reaction-center protein is highly susceptible to photodamage [5,12,13]. Nevertheless, damaged PSII can be rapidly repaired through the D1 turnover cycle [12,14], and this PSII photoinhibition–repair cycle in turn protects PSI from irreversible damage [15,16]. By contrast, PSI was historically regarded as resistant to photoinhibition, but it can be preferentially inhibited during chilling, especially under illumination [17,18,19]. Owing to the slow turnover of its core proteins, once PSI is damaged, its recovery is extremely slow [15,18]. Moreover, because the electron transport chain functions as an integrated whole, chilling-induced inhibition of carbon assimilation leads to over-reduction in the PSI acceptor side and the generation of reactive oxygen species [18,20], posing a risk of PSI photoinhibition.

It is widely recognized that the site of chilling-induced photoinhibition depends on light intensity: PSII is preferentially photoinhibited under strong light, whereas PSI is preferentially photoinhibited under weak light [21]. For example, in cucumber (*Cucumis sativus*) exposed to 4 °C and 220 µmol·m^−2^·s^−1^, PSI was photoinhibited more severely than PSII [17]. Takeuchi et al. further reported that when two cucumber cultivars differing in chilling tolerance were exposed to 4 °C and 180–250 µmol·m^−2^·s^−1^, PSII photoinhibition was comparable between the cultivars, whereas PSI was severely photoinhibited in the chilling-sensitive cultivar but only mildly in the chilling-tolerant one [20]. Huang et al. also reported that chilling at 4 °C and 250 µmol·m^−2^·s^−1^ induced PSII photoinhibition in all four tropical tree species examined (*Khaya ivorensis*, *Pometia tomentosa*, *Dalbergia odorifera*, and *Erythrophleum guineense*), whereas PSI photoinhibition was detected only in *E. guineense* [22]. Taken together, these observations demonstrate that both PSII and PSI can undergo photoinhibition under illumination at chilling temperature, but their relative sensitivity differs depending on plant species, temperature, and light intensity [18,23,24,25,26].

To counteract chilling-induced photoinhibition, plants activate multiple photoprotective pathways that dissipate or reallocate excess excitation energy, including xanthophyll cycle-dependent thermal dissipation (non-photochemical quenching, NPQ), cyclic electron flow (CEF), and the water-water cycle (WWC) [18,27,28,29]. For example, NPQ was upregulated in mulberry (*Morus alba*) under chilling conditions [30]. Long-term chilling at 4 °C markedly enhanced CEF in the chilling-tolerant orchid *Cymbidium tracyanum*, whereas such upregulation was limited in the chilling-sensitive *C. sinense* [31]. In the medicinal orchid *Dendrobium officinale*, the WWC was inactivated at 4 °C and was partially compensated for by enhanced CEF [32]. These observations indicate that the photoprotective strategy deployed by plants under chilling stress varies among species and stress conditions.

The effects of LSC on the photosynthetic apparatus of crops have been investigated to some extent. However, most of these studies have focused on staple crops such as wheat and rice, whereas research on medicinal plants, particularly herbaceous medicinal plants such as McJXR, remains scarce. Moreover, the majority of previous studies were conducted under artificially controlled chilling conditions. In contrast, a natural LSC event involves a combination of abrupt temperature drops and natural light fluctuations; its effects on the photosynthetic apparatus may therefore differ from those of pure chilling treatment. In particular, whether the post-LSC light environment influences photosynthetic recovery remains unexplored in medicinal plants such as McJXR. Nevertheless, the light environment during the recovery period is critical for the restoration of photosynthetic function. For example, high light after chilling was found to delay photosynthetic recovery in tomato (*Solanum lycopersicum*) [33]. In this study, we exposed McJXR seedlings to a natural LSC event in the spring of 2025. During the recovery period, the seedlings were divided into two groups and subjected to shading (R_LL_) or full sunlight (R_HL_). Chlorophyll fluorescence parameters of PSII and PSI were measured synchronously with a Dual-PAM-100/F. We aimed to characterize the responses of PSII and PSI function to LSC and subsequent recovery light intensity.

## 2. Results

### 2.1. Effects of LSC and Recovery Light Intensity on Fv/Fm and Pm

**Figure 1:**
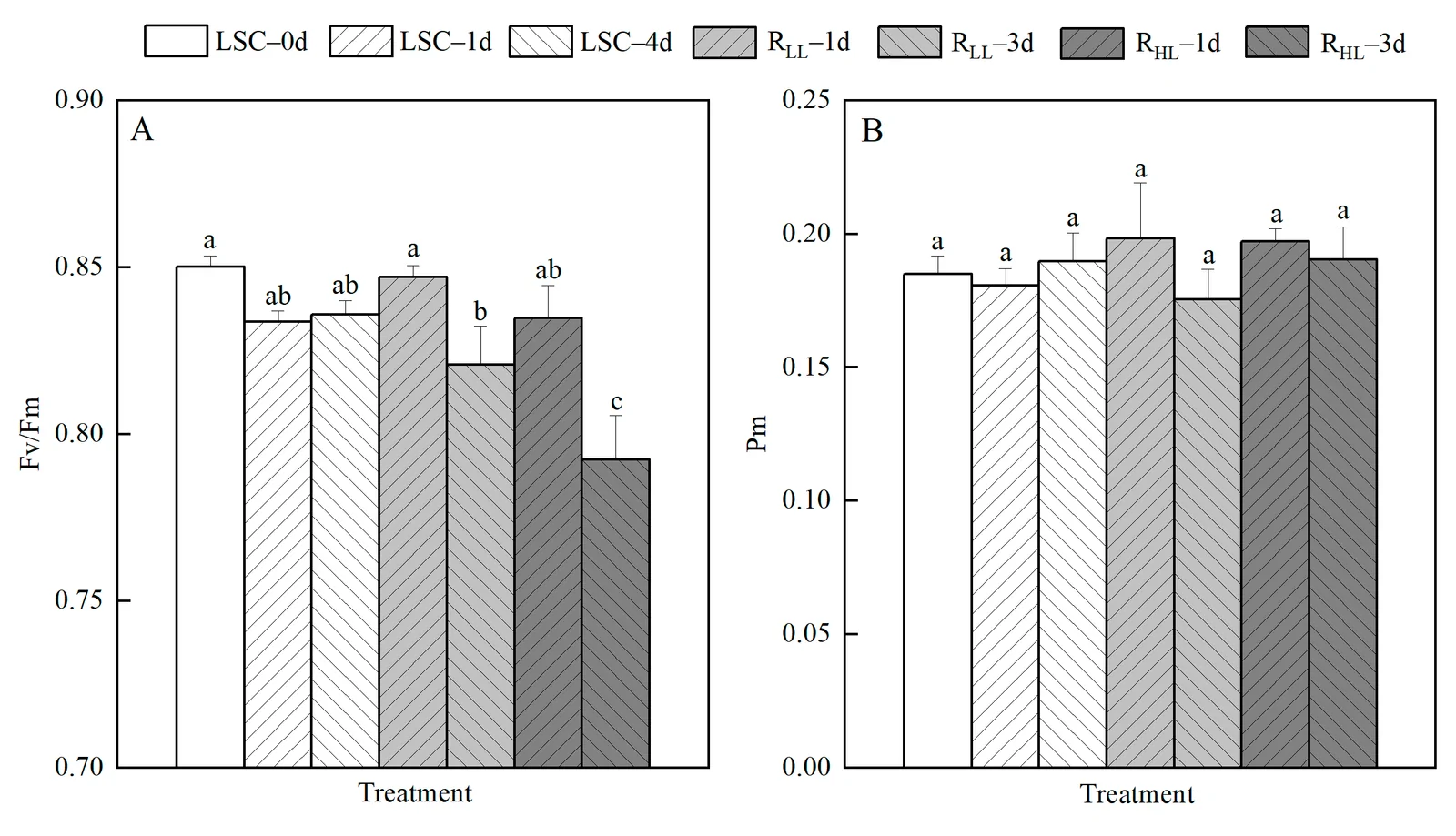
Effects of late spring coldness and recovery light intensity on the maximum photochemical efficiency of photosystem II (Fv/Fm) (**A**) and the maximum P700 signal (Pm) (**B**) in *Mosla chinensis* Maxim. cv. Jiangxiangru seedlings. The bars with different lowercase letters are significantly different among treatments (*p* < 0.05). Values are means ± SE (*n* = 5).

During the LSC period, the maximum photochemical efficiency of PSII (Fv/Fm) in McJXR seedlings showed a slight but non-significant decrease relative to the pre-stress level (LSC-0d) (Figure 1A). On the first day of recovery, Fv/Fm remained essentially unchanged under both treatments. By the third day of recovery, however, Fv/Fm had decreased significantly under both the shading (R_LL_, daily maximum light intensity of approximately 500 µmol·m^−2^·s^−1^) and the full-sunlight (R_HL_, daily maximum light intensity of approximately 1100 µmol·m^−2^·s^−1^) treatments, with a significantly greater decline under full sunlight than under shading (Figure 1A). In contrast, the maximum P700 signal (Pm) showed no significant changes throughout the LSC and recovery periods under either treatment (Figure 1B).

### 2.2. Effects of LSC and Recovery Light Intensity on Energy Partitioning of PSII and PSI

**Figure 2:**
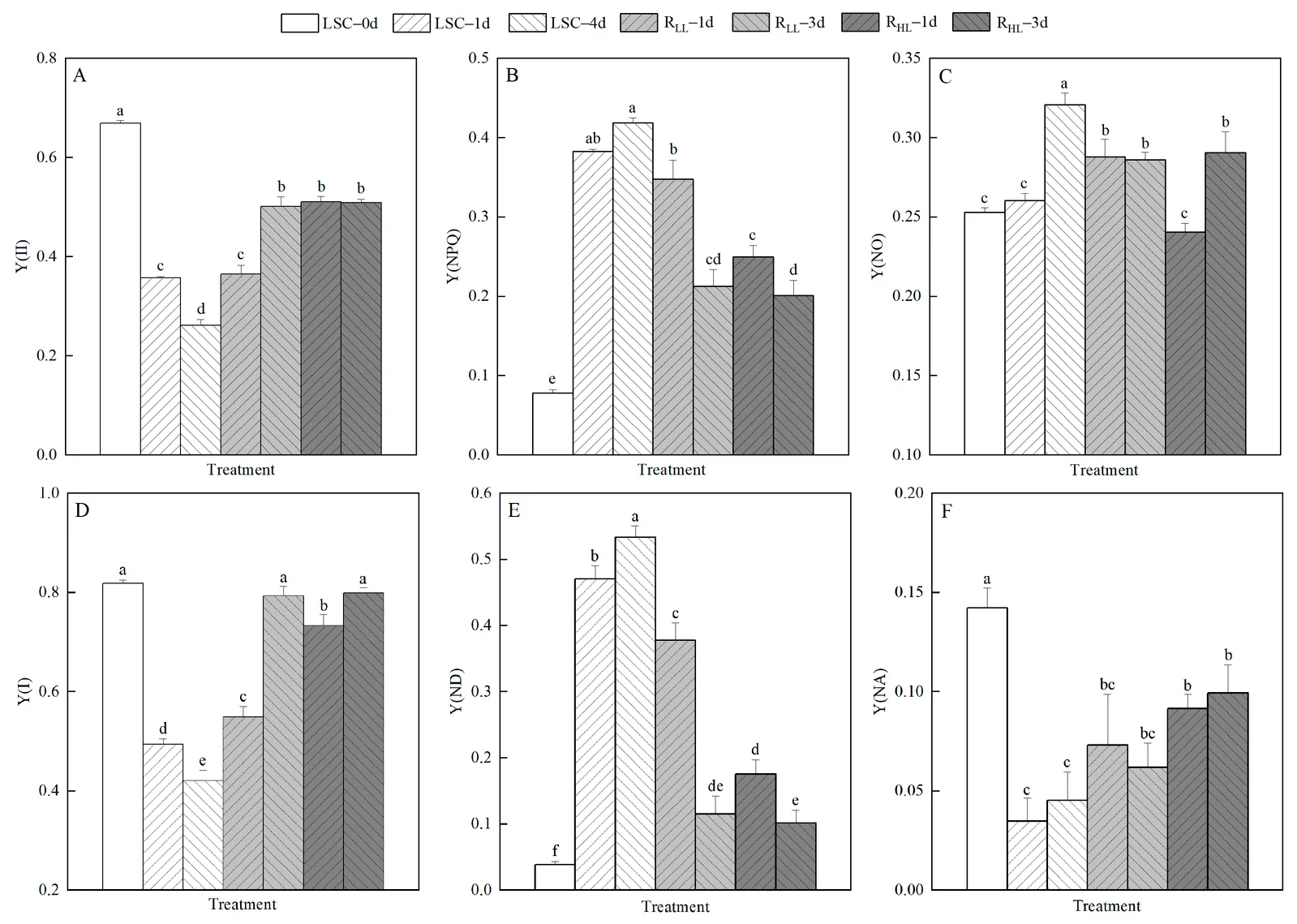
Effects of late spring coldness and recovery light intensity on the energy partitioning of photosystem II and photosystem I in *Mosla chinensis* Maxim. cv. Jiangxiangru seedlings. Photochemical quantum yields of PSII (Y(II)) (**A**), quantum yield of regulated energy dissipation in PSII (Y(NPQ)) (**B**), quantum yield of non-regulated energy dissipation in PSII (Y(NO)) (**C**), photochemical quantum yields of PSI (Y(I)) (**D**), quantum yields of non-photochemical energy dissipation due to donor-side (Y(ND)) (**E**), quantum yields of non-photochemical energy dissipation due to acceptor-side (Y(NA)) (**F**).The bars with different lowercase letters are significantly different among treatments (*p* < 0.05). Values are means ± SE (*n* = 5).

During the LSC period, a progressive decline in the photochemical quantum yield of PSII (Y(II)) was accompanied by a sustained increase in the quantum yield of regulated energy dissipation in PSII (Y(NPQ)), whereas the quantum yield of non-regulated energy dissipation in PSII (Y(NO)) remained largely stable until it rose significantly at LSC-4d (Figure 2A–C). Relative to the LSC-0d level, Y(II) decreased by 61.0% at LSC-4d, whereas Y(NPQ) and Y(NO) increased by 5.4-fold and 1.3-fold, respectively. During recovery, Y(II) increased significantly under both treatments relative to LSC-4d but remained significantly below the LSC-0d level. On the first day of recovery, Y(II) was significantly lower under shading than under full sunlight; by the third day, however, this difference was no longer evident. Y(NPQ) declined progressively under both treatments during recovery; it was significantly lower under full sunlight than under shading on the first day of recovery, but this difference ceased to be significant by the third day. On the first day of recovery, Y(NO) decreased significantly under both treatments relative to LSC-4d, with a significantly greater decrease under full sunlight than under shading. By the third day, Y(NO) remained stable under shading but had increased significantly under full sunlight, with no significant difference between the two treatments.

During the LSC period, the photochemical quantum yield of PSI (Y(I)) declined by 39.7% and 48.6% relative to the LSC-0d level on the first and fourth days, respectively (Figure 2D–F). Concurrently, the quantum yields of non-photochemical energy dissipation due to donor-side (Y(ND)) increased to 12.1-fold and 13.8-fold of the LSC-0d level on the first and fourth days, respectively. By contrast, the quantum yields of non-photochemical energy dissipation due to acceptor-side (Y(NA)) decreased significantly by 75.5% on the first day of LSC and subsequently remained stable. During recovery, Y(I) recovered significantly under both treatments relative to LSC-4d; it was significantly higher under full sunlight than under shading on the first day of recovery, and both treatments had returned to the LSC-0d level by the third day. Y(ND) declined progressively during recovery and was significantly lower under full sunlight than under shading on the first day; by the third day, although Y(ND) remained significantly above the LSC-0d level, the difference between the two treatments had ceased to be significant. During recovery, Y(NA) increased significantly under full sunlight relative to LSC-4d but showed no significant change under shading; no significant difference was detected between the two treatments, and Y(NA) remained significantly below the LSC-0d level under both treatments.

### 2.3. Effects of LSC and Recovery Light Intensity on qP, NPQ, and CEF

**Figure 3:**
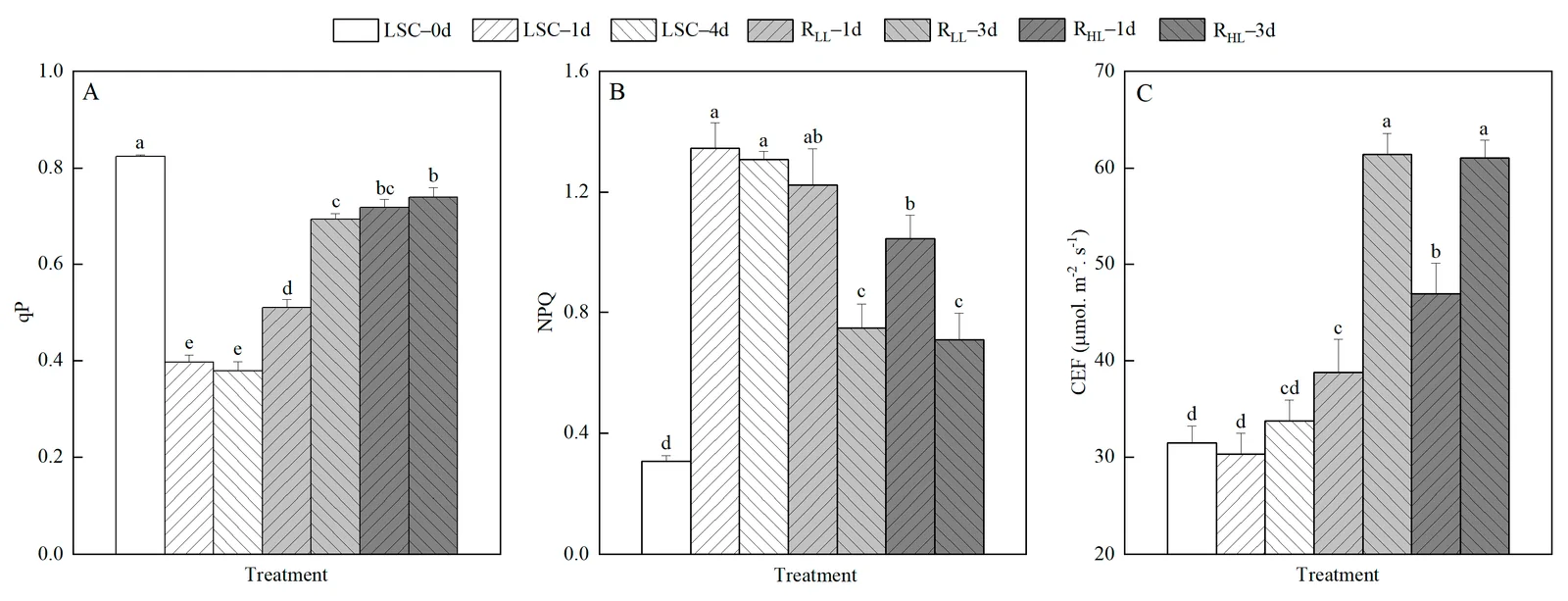
Effects of late spring coldness and recovery light intensity on the photochemical quenching coefficient (qP) (**A**), non-photochemical quenching (NPQ) (**B**), and the cyclic electron flow (CEF) (**C**) in *Mosla chinensis* Maxim. cv. Jiangxiangru seedlings. The bars with different lowercase letters are significantly different among treatments (*p* < 0.05). Values are means ± SE (*n* = 5).

During the LSC period, the photochemical quenching coefficient (qP) decreased sharply, whereas NPQ increased markedly (Figure 3A,B). On the first day of LSC, qP decreased by 51.9% relative to the LSC-0d level, whereas NPQ rose to 4.4-fold the LSC-0d level; both parameters subsequently remained stable. Meanwhile, CEF showed no significant change during the LSC period (Figure 3C).

During recovery, qP increased significantly under both treatments relative to LSC-4d but did not return to the LSC-0d level. Under shading, qP recovered to 61.9% and 84.0% of the LSC-0d level on the first and third days of recovery, respectively, compared with 87.1% and 89.8% under full sunlight. On the first day of recovery, NPQ under shading showed no significant difference from the LSC-4d level, whereas NPQ under full sunlight decreased significantly. By the third day of recovery, NPQ had declined further under both treatments and remained above the LSC-0d level, with no significant difference between the two treatments. During recovery, CEF increased markedly under both treatments, rising to 123.2% and 195.1% of the LSC-0d level under shading and to 149.0% and 193.8% under full sunlight on the first and third days of recovery, respectively. CEF was significantly higher under full sunlight than under shading on the first day of recovery, whereas no significant difference between the two treatments was observed on the third day.

### 2.4. Effects of LSC and Recovery Light Intensity on the Light Response of Photosynthetic Electron Transport

**Figure 4:**
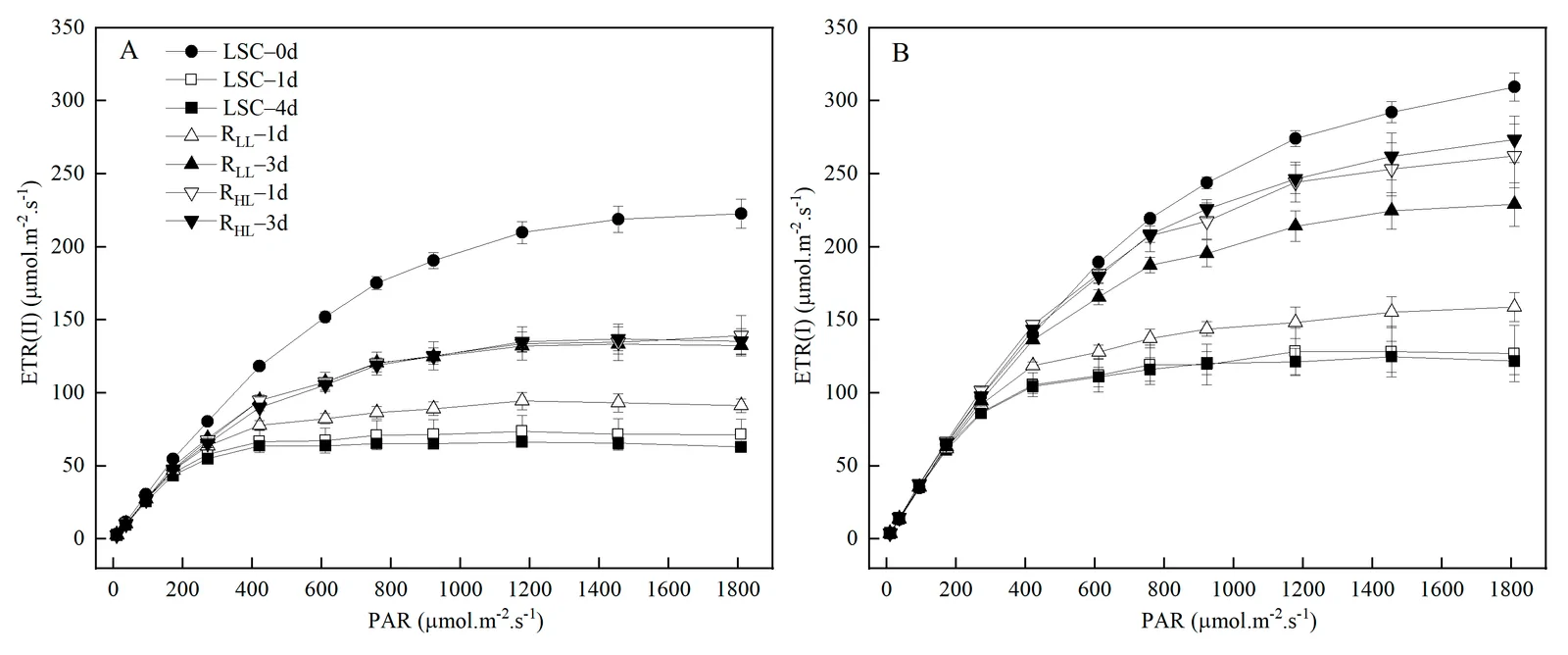
Effects of late spring coldness and recovery light intensity on the rapid light curves of the electron transport rates through photosystem II (ETR(II)) (**A**) and photosystem I (ETR(I)) (**B**) in *Mosla chinensis* Maxim. cv. Jiangxiangru seedlings. Values are means ± SE (*n* = 5).

During the LSC period, the electron transport rates through PSII (ETR(II)) and PSI (ETR(I)) decreased significantly relative to the LSC-0d level, and their light-response curves were similar between LSC-1d and LSC-4d (Figure 4). Under full sunlight, both ETR(II) and ETR(I) recovered markedly by the first day of recovery, with no further significant change by the third day. Under shading, by contrast, ETR(II) and ETR(I) increased progressively over the recovery period. On the first day of recovery, both ETR(II) and ETR(I) were significantly higher under full sunlight than under shading. By the third day, ETR(II) no longer differed significantly between the two treatments, whereas ETR(I) remained significantly higher under full sunlight than under shading.

**Figure 5:**
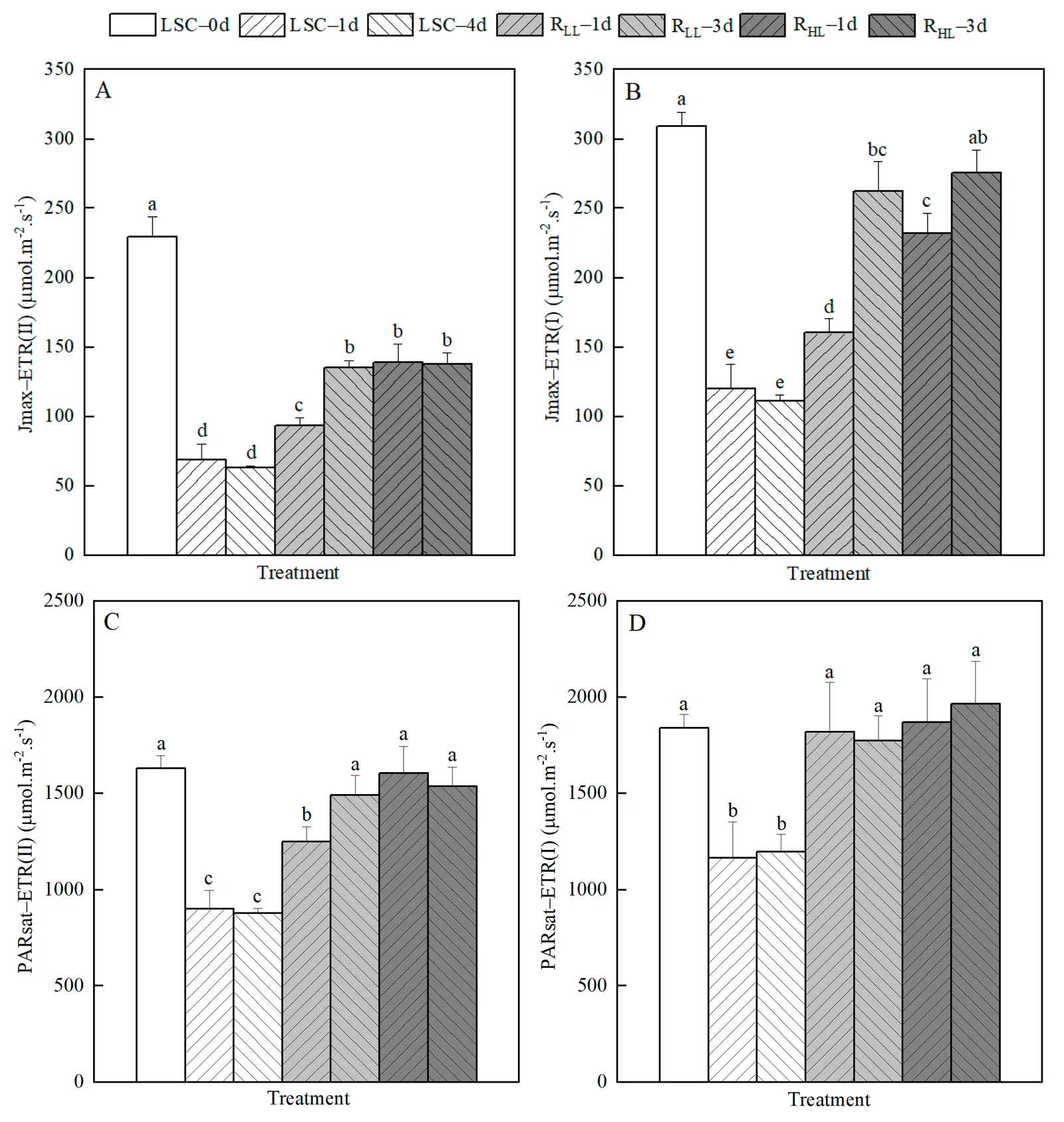
Effects of late spring coldness and recovery light intensity on the maximum electron transport rate (Jmax-ETR(II) (**A**) and Jmax-ETR(I) (**B**)) and the saturating light intensity (PARsat-ETR(II) (**C**) and PARsat-ETR(I) (**D**)) in *Mosla chinensis* Maxim. cv. Jiangxiangru seedlings. The bars with different lowercase letters are significantly different among treatments (*p* < 0.05). Values are means ± SE (*n* = 5).

During the LSC period, the maximum electron transport rate (Jmax-ETR(II) and Jmax-ETR(I)) and the corresponding saturating light intensity (PARsat-ETR(II) and PARsat-ETR(I)) decreased significantly (Figure 5). On the first day of LSC, Jmax decreased by 70.1% for PSII and 61.1% for PSI, whereas PARsat decreased by 44.7% and 36.7%, respectively; thereafter, both parameters remained stable. During recovery, Jmax and PARsat increased significantly under both treatments relative to LSC-4d. On the first day of recovery, Jmax was significantly higher under full sunlight than under shading. By the third day, Jmax did not differ significantly between the two treatments for either photosystem, although only Jmax-ETR(I) under full sunlight had recovered to the LSC-0d level. For PARsat, recovery to the LSC-0d level occurred by the first day of recovery in all cases except for PARsat-ETR(II) under shading, which recovered only by the third day.

## 3. Discussion

Natural late spring coldness events frequently threaten spring crops. In the Jianghuai region of China, LSC events with mean temperatures of about 4–5 °C and minima below −4 °C caused severe frost damage and yield losses in wheat [34]. In Hungary, natural cold events during the spring seedling stage of rice, with mean temperatures of about 14–15 °C and minima around 9 °C over two weeks, significantly reduced the photochemical performance of PSII and caused chlorophyll degradation and stunted seedling growth, with chilling-sensitive genotypes being markedly more affected than tolerant ones [35]. These findings demonstrate that natural spring cold events can induce photoinhibition or severe photosynthetic damage in spring crops such as rice and wheat. Fv/Fm and Pm reflect the maximum photochemical efficiency of PSII and the content of active PSI complexes, respectively, and are important parameters for evaluating PSII and PSI photoinhibition [18,31,36,37]. In the present study, however, neither Fv/Fm nor Pm decreased significantly during the LSC period (Figure 1A,B), which indicates that the natural LSC event did not induce photoinhibition of either photosystem in McJXR. This difference may be related to the specific conditions of the cold event: the cold spell experienced by McJXR (4.5–12.8 °C for four days, without sub-zero temperatures) was a moderate chilling event, in contrast to the freezing temperatures that damaged wheat [34]. Nevertheless, the marked declines in Y(II), Y(I), and qP (Figure 2A,D and Figure 3A) suggest that the seedlings perceived and responded to the stress, and the functional activities of the two photosystems may have been inhibited to different extents.

Photosynthetic electron transport is mediated by the tandem operation of PSII and PSI, and the coordinated function of the two photosystems is essential for its smooth progression [38]. The two photosystems exhibit differential sensitivity to chilling, and their relative susceptibility is strongly influenced by plant species and stress conditions [18,23]. The responses of the two photosystems in McJXR seedlings during the natural LSC event were largely consistent with this general pattern. During the LSC period, Y(II) and Y(I), which represent the efficiencies of converting absorbed light energy into chemical energy by PSII and PSI, respectively [39,40], decreased continuously, and the decrease was significantly greater for Y(II) than for Y(I) (Figure 2A,D). Likewise, ETR(II) and ETR(I) decreased significantly at all light intensities (Figure 4), and the reductions in Jmax and PARsat were larger for ETR(II) than for ETR(I) (Figure 5). These results indicate that the LSC event suppressed the electron transport and light-use capacity of PSII more strongly than those of PSI. Consistently, qP, which reflects the fraction of open PSII reaction centers [36], dropped by 51.9% on the first day of LSC (Figure 3A), indicating that a large fraction of PSII reaction centers were closed under chilling. This preferential suppression of PSII is in line with observations in other species, including tomato leaves chilled at 8 °C [41] and tropical tree species chilled at 4 °C under moderate light [22]. The relative sensitivity of the two photosystems, however, is not fixed but depends on light intensity and the severity of cold stress [18,21]; when the temperature approaches the freezing point, the site of photoinhibition can shift toward PSI, as demonstrated in cucumber chilled at 4 °C, in which PSI was damaged more severely than PSII [17]. The mechanistic basis of this differential sensitivity lies in the distinct regulatory strategies of the two photosystems. PSII relies on the continuous photodamage–repair cycle of its D1 reaction-center protein, which is metabolically demanding and strongly slowed at low temperatures [15,42]; in addition, chilling-induced inhibition of carbon assimilation reduces the downstream demand for electrons, promoting the closure of PSII reaction centers and a rapid decline in Y(II) [5]. By contrast, PSI is protected mainly by preventive mechanisms, most notably the oxidation of P700, which dissipates excess excitation energy before it can damage the PSI reaction center [18]. In the present study, this protection remained effective under moderate chilling; PSI was therefore functionally constrained but structurally intact [15,18].

Chilling imposes an excess of excitation energy on PSII because the consumption of ATP and NADPH by carbon assimilation is strongly suppressed, and plants must dissipate this excess energy through photoprotective pathways to prevent oxidative damage [27,43]. Xanthophyll cycle-dependent thermal dissipation, generally quantified as NPQ, converts excess excitation energy absorbed by the PSII antenna into heat and is one of the most rapid photoprotective responses [27,44,45]. In the present study, the rapid and pronounced upregulation of NPQ (Figure 3B) indicates that regulated thermal dissipation was effectively activated in McJXR seedlings, allowing the excess excitation energy arising from the closure of PSII reaction centers (the decline in qP, Figure 3A) to be safely dissipated as heat. Meanwhile, Y(NPQ) increased sharply on the first day of the LSC event and remained elevated throughout the LSC period, whereas Y(NO) increased only slightly by LSC-4d (Figure 2B,C), which also indicates that the excess excitation energy was dissipated predominantly through the regulated pathway rather than being channeled into the non-regulated route that carries a risk of photodamage [39]. Enhanced thermal dissipation under chilling has also been observed in mulberry [30] and wheat [46]. These results indicate that the rapid activation of thermal dissipation protected PSII from irreversible damage during the LSC event, although at the cost of a marked reduction in PSII photochemical efficiency.

The core proteins of PSI turn over slowly, and once PSI is photoinhibited, its recovery is much slower than that of PSII [15,18]. The downregulation of PSII, which restricts the electron supply to PSI, is therefore considered a primary protective mechanism against PSI photodamage [15]. In the present study, the decline in Y(I) during the LSC period was accompanied by a pronounced increase in Y(ND) and a decrease in Y(NA) (Figure 2D–F), indicating that PSI was limited predominantly on the donor side [40]. The elevated Y(ND) was a direct consequence of the downregulation of PSII, which limited the electron supply from PSII to PSI and prevented the over-reduction in the PSI acceptor side, thereby protecting PSI from photodamage [15]. In tobacco (*Nicotiana tabacum*), chilling was also found to inhibit PSII activity, which limited electron transport from PSII to PSI and thereby alleviated PSI photodamage [16]. CEF also contributes to the protection of PSI by recycling electrons from the PSI acceptor side back to the plastoquinone pool and thereby alleviating its over-reduction [47]. In McJXR seedlings, however, CEF showed no significant change during the LSC period (Figure 3C), indicating that PSI was protected mainly through the downregulation of PSII rather than through enhanced cyclic electron transport.

The recovery of the photosynthetic apparatus after chilling depends not only on the severity and duration of the preceding stress but also on the light conditions during the recovery period [33]. On the one hand, excessively strong light can exceed the utilization capacity of the still-recovering photosynthetic apparatus, aggravating photoinhibition and even causing irreversible damage [17]. On the other hand, a certain level of light is required for the repair of photodamaged PSII, which involves the light-dependent de novo synthesis of the D1 reaction-center protein [42,48]. The higher light intensity of the full-sunlight treatment during recovery aggravated the photoinhibition of PSII, as indicated by the greater decline in Fv/Fm (Figure 1A) and as also reported in tomato [41]. Nevertheless, a certain level of light was beneficial for the restoration of the photochemical functions of both photosystems, as shown by the more rapid recovery of Y(II), Y(I), and qP under the full-sunlight treatment than under the shading treatment on the first day of recovery (Figure 2A,D and Figure 3A). Furthermore, by the third day of recovery, Y(II), qP, and Jmax-ETR(II) did not recover fully under either treatment (Figure 2A, Figure 3A and Figure 5A). By contrast, Y(I) recovered completely under both treatments (Figure 2D), whereas Jmax-ETR(I) recovered completely only under the full-sunlight treatment (Figure 5B). These results indicate that PSII was more sensitive to the LSC event and that its suppression was more persistent than that of PSI. This difference may reflect the fact that, unlike PSII, PSI requires no light-dependent repair once it has been spared from photodamage. In addition, the slower recovery of PSII function may be partly related to the different dynamics of NPQ and CEF during the recovery period. During recovery, NPQ decreased rapidly under both treatments, whereas CEF increased markedly (Figure 3B,C). The rapid decrease in NPQ, although favoring the reallocation of light energy to photochemistry, may have exposed the still-repairing PSII to excess excitation energy and delayed its recovery. It should be noted that the two recovery regimes differed not only in light intensity but also, potentially, in other microclimatic variables, since a shade net may modify air temperature, humidity and wind speed beneath it; the two regimes therefore represent contrasting light environments as a whole rather than a pure gradient of light intensity. Within this contrast, however, light intensity was the variable deliberately imposed, and its difference was by far the largest (approximately two-fold in maximum midday light intensity). Moreover, the two treatments were applied simultaneously at the same site and within a similar temperature range (daily maximum 21.3–28.9 °C), and the observed pattern—an aggravated decline in Fv/Fm together with a more pronounced recovery of Y(II), Y(I) and qP under full sunlight—matches the well-established dual role of light during recovery, rather than an effect of temperature or humidity. Light intensity is therefore considered the most likely principal factor, although a minor contribution of other microclimatic variables cannot be excluded. Taken together, these results indicate that the full-sunlight treatment accelerated the recovery of the photochemical activity of both photosystems relative to the shading treatment, and that the differential recovery of PSII and PSI was associated with the differential dynamics of NPQ and CEF.

It should be noted that a natural LSC event involves coupled changes in temperature and light, and the responses observed here therefore reflect the response of the seedlings to a cold, low-light environment as a whole. Nevertheless, the downregulation of the two photosystems appears to have been driven mainly by low temperature: although the light available during the LSC period was low, the seedlings still displayed pronounced signs of excess excitation pressure, indicating that their capacity to utilize absorbed light was restricted by low temperature rather than by the light regime [5]. Because the present study is based on a single natural event, the conclusions should be regarded as specific to the conditions of this event rather than as general responses of McJXR to LSC. Even so, the study of this single natural event has its own value. The event represents a real field scenario, and the observations reflect the authentic responses of the plants under field conditions. To our knowledge, this is the first report on the functional responses of both photosystems of McJXR to a natural LSC event, providing baseline data and testable hypotheses for future studies. Further validation involving LSC events across multiple seasons, together with controlled-environment experiments under defined temperature and light regimes, is needed to establish the generality of these findings and to deepen our understanding of the responses of McJXR to LSC stress.

## 4. Materials and Methods

### 4.1. Site Description

**Figure 6:**
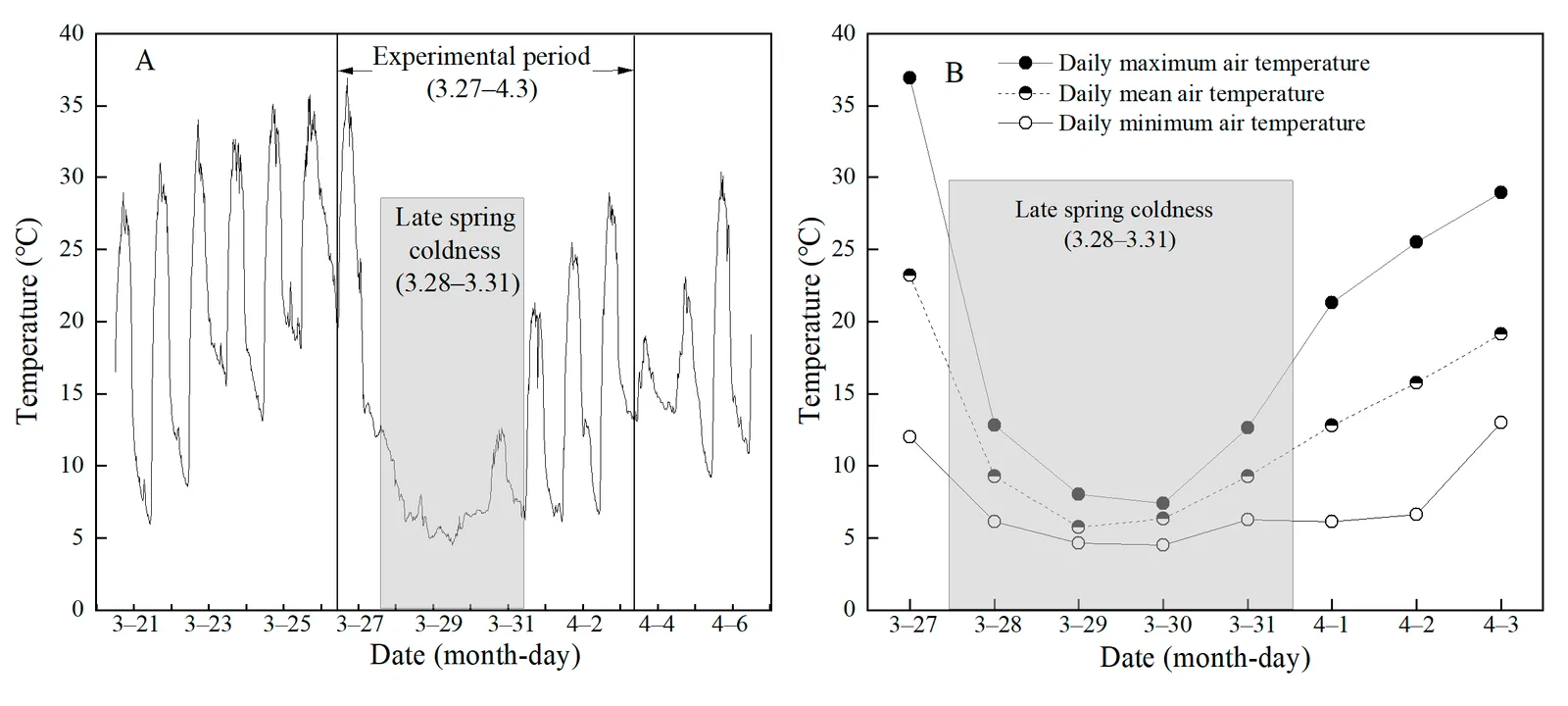
Changes in daily air temperature during experimental period. (**A**) The continuous diurnal dynamics of air temperature during the experimental period and (**B**) the daily maximum, mean, and minimum air temperature from 27 March to 3 April.

The experiment was conducted at Jinggangshan University, Ji’an, Jiangxi Province, China (115.02° E, 27.11° N, 80 m above sea level), which belongs to a typical mid-subtropical humid monsoon climate zone. The experiment was performed from late March to early April 2025, and the changes in air temperature during this period are shown in Figure 6A. Based on this record, an LSC event was identified from 28 to 31 March. During the LSC event, the daily maximum/minimum air temperature dropped sharply from 36.9 °C/12.0 °C on 27 March to 12.8 °C/6.1 °C on 28 March, and then remained at 7.4–12.8 °C (maximum) and 4.5–6.3 °C (minimum) (Figure 6B). After the LSC event, the temperature rose to 21.3–28.9 °C (maximum) and 6.1–13.0 °C (minimum) during the recovery period (1–3 April). The LSC period was overcast and rainy, with a maximum midday light intensity of approximately 300 µmol·m^−2^·s^−1^, whereas the recovery period was sunny, with a maximum midday light intensity of approximately 1100 µmol·m^−2^·s^−1^. Air temperature was recorded continuously at 15 min intervals at 12 cm above the ground surface using a TMS soil temperature and moisture datalogger (TOMST s.r.o., Prague, Czech Republic) placed in the open field, and light intensity was measured with a LI-250A light meter equipped with a quantum sensor (LI-COR, Lincoln, NE, USA).

### 4.2. Experimental Design

Seeds of McJXR were obtained from Jinbei Agricultural Science and Technology Development Co., Ltd. (Fenyi County, Xinyu City, Jiangxi Province, China). In early January 2025, the seeds were sown in seedling trays filled with a commercial peat substrate (Hawita Gruppe GmbH, Vechta, Germany; distributed in China by Hongyue Horticultural Corporation) composed of 70% white peat and 30% black peat (fiber length 0–5 mm, pH 5.5–6.0 and EC < 0.5 mS·cm^−1^) and germinated in an artificial climate chamber maintained at 25/20 °C (day/night) with a light intensity of 200–300 µmol·m^−2^·s^−1^, in which all seedlings were grown until 20 March. After emergence, the seedlings were irrigated with half-strength Enshi nutrient solution once a week in the trays for approximately one month. Subsequently, the seedlings were transplanted into plastic pots (12 cm in diameter, 14 cm in height) filled with the same substrate and irrigated with full-strength Enshi nutrient solution every 3–4 days. The nutrient solution was a commercial preparation of ‘Enshi’ nutrient solution (Shandong Fengben Biotechnology Co., Ltd., Weifang, China). The composition of the full-strength solution was as follows (mg·L^−1^): Ca(NO_3_)_2_·4H_2_O 950, KNO_3_ 810, MgSO_4_·7H_2_O 500, NH_4_H_2_PO_4_ 155, H_3_BO_3_ 3, ZnSO_4_·7H_2_O 0.22, MnSO_4_·5H_2_O 2, CuSO_4_·5H_2_O 0.05, Na_2_MoO_4_ 0.02 and NaFe-EDTA 25 [49,50]. The same nutrient solution was supplied to all plants throughout the experiment. On 20 March, uniform seedlings approximately 20–23 cm in height were transferred to an open outdoor area for acclimation and continued to receive the same nutrient solution.

The experiment was conducted from 27 March to 3 April 2025. A total of 20 pots of uniform seedlings were first subjected to the natural LSC event in the open outdoor area. On the evening of 31 March, the 20 pots were randomly divided into two groups of 10 pots each, and two recovery light regimes were established to represent the contrasting light conditions that commonly occur after an LSC event. One group was placed under a single layer of sunshade net (shading treatment; low light during recovery, R_LL_) to mimic the overcast or cloudy conditions that may follow an LSC event, and the other was kept in the open area (full-sunlight treatment; high light during recovery, R_HL_) as the natural, unshaded field condition. The daily maximum light intensities under the shading and full-sunlight treatments were approximately 500 and 1100 µmol·m^−2^·s^−1^, respectively. Chlorophyll fluorescence was measured in the morning on 27 March (LSC-0d), 28 March (LSC-1d), 31 March (LSC-4d), 1 April (R_LL_-1d and R_HL_-1d), and 3 April (R_LL_-3d and R_HL_-3d). On each measurement date, five pots (*n* = 5) were randomly and independently selected for measurement and were returned to their original position afterwards.

### 4.3. Determination of Chlorophyll Fluorescence Parameters

Chlorophyll fluorescence signals of PSII and PSI were recorded simultaneously using the “P700+Fluo” mode of a Dual-PAM-100/F fluorometer (Heinz Walz GmbH, Effeltrich, Germany). Before measurement, leaves were dark-adapted for 30 min. First, the chlorophyll fluorescence induction curve was recorded in the “Slow Kinetics” mode: after determining the minimum (Fo) and maximum (Fm) fluorescence of PSII and Pm in the dark-adapted state, actinic light of 508 µmol·m^−2^·s^−1^ was applied, and chlorophyll fluorescence parameters were recorded every 20 s for an induction period of 240 s, when the induction curve had become stable; the values recorded at the last time point were used for analysis. Then, rapid light curves of electron transport through PSII and PSI were measured in the “Light Curve” mode by applying actinic light increasing stepwise from 10 to 1809 µmol·m^−2^·s^−1^, with each light intensity maintained for 20 s before recording.

The following parameters were obtained directly from the instrument software: Y(II), Y(NPQ), Y(NO), qP, NPQ, Y(I), Y(ND), Y(NA), ETR(II) and ETR(I). Fv/Fm was calculated as (Fm—Fo)/Fm, and CEF was calculated as ETR(I)—ETR(II) [51]. The rapid light curves of ETR(II) and ETR(I) were fitted using the mechanistic model of the light response of photosynthetic electron transport [52], from which Jmax and PARsat for PSII and PSI were calculated.

### 4.4. Statistical Analysis

Data were analyzed using SPSS 18.0 software (SPSS Inc., Chicago, IL, USA). Each treatment × measurement time combination was treated as a separate group. One-way analysis of variance (ANOVA) with the least significant difference (LSD) test was used to compare differences among groups. All data are presented as means ± standard error (SE), and differences were considered statistically significant at *p* < 0.05. Figures were prepared using Origin 2021 (OriginLab Corporation, Northampton, MA, USA).

## 5. Conclusions

In summary, the natural LSC event did not induce photoinhibition of either photosystem, although PSII activity was significantly more sensitive to chilling than PSI. During the LSC period, the photoprotection of PSII depended mainly on the rapid activation of thermal dissipation, whereas PSI was protected mainly through the downregulation of PSII function. During the recovery period, the full-sunlight treatment accelerated the recovery of the photochemical activity of both photosystems relative to the shading treatment, although it also increased the degree of PSII photoinhibition, and the recovery capacity of PSI was superior to that of PSII. The rapid decline in thermal dissipation, together with the marked increase in cyclic electron flow during recovery, may be an important reason for the slower recovery of PSII than PSI.

## Data Availability

The data supporting our findings and analyses are included in the article itself. Readers can access this data by referring to the article.
